# Efficacy and predictors of cognitive stimulation therapy combined with pharmacotherapy for mild-to-moderate Alzheimer’s disease: a randomized controlled trial

**DOI:** 10.3389/fpsyt.2026.1840039

**Published:** 2026-06-26

**Authors:** Li Cai, Xinmin Zhao, Chunmei Liao, Qin Liu, Shu Hu, Min Huang

**Affiliations:** Department of Geriatrics, Chongqing Mental Health Center, Chongqing, China

**Keywords:** Alzheimer’s disease, cognitive reserve, Cognitive Stimulation Therapy, randomized controlled trial, dementia, cognitive intervention, cognitive training, physical activity

## Abstract

**Introduction:**

Alzheimer’s disease (AD) is associated with progressive cognitive decline, functional impairment, and reduced quality of life. Although pharmacological treatments such as cholinesterase inhibitors and memantine are commonly used, their clinical benefits remain limited and heterogeneous. Cognitive stimulation therapy (CST) may provide additional benefits when combined with standard pharmacotherapy. This randomized controlled trial (RCT) aimed to evaluate the clinical efficacy of modified CST combined with standard drug therapy on cognitive function, activities of daily living, and quality of life in patients with mild-to-moderate AD and to explore key predictors of CST efficacy using a multivariate regression model.

**Methods:**

This evaluator-blinded randomized controlled trial enrolled 80 patients with mild-to-moderate Alzheimer’s disease (AD), who were randomly assigned in a 1:1 ratio to either the modified CST plus standard pharmacotherapy group (study group, n = 40) or the standard pharmacotherapy-alone group (control group, n = 40).The modified CST program comprised 14 weekly 45-minute sessions. The primary endpoint was the change in the Alzheimer’s Disease Assessment Scale–Cognitive Subscale (ADAS-Cog) score from baseline to post-intervention. Secondary measures included the Activities of Daily Living (ADL) scale and the Quality of Life in Alzheimer’s disease (QOL-AD) questionnaire. Data were analyzed using an intention-to-treat (ITT) approach. Independent predictors of treatment efficacy were identified using a two-stage screening strategy (univariate screening and stepwise regression).

**Results:**

A total of 75 patients completed the study, and 80 were included in the ITT analysis. After 14 weeks of intervention, baseline-adjusted ANCOVA showed that the study group had significantly better post-intervention ADAS-Cog scores than the control group. The adjusted mean difference in ADAS-Cog score was -3.28 points (95% CI: -3.72 to -2.83; P < 0.001), favoring the study group. Significant baseline-adjusted between-group differences were also observed for ADL (adjusted mean difference = -4.93, 95% CI: -8.39 to -1.47; P = 0.006) and QOL-AD (adjusted mean difference = 2.69, 95% CI: 1.01 to 4.37; P = 0.002), both favoring the study group. Higher years of education (β = -0.54, P < 0.001), regular physical activity (β = -0.28, P = 0.003), higher baseline Mini-Mental State Examination (MMSE) scores (β = -0.22, P = 0.001), and active hobbies (β = -0.20, P = 0.002) were significant independent predictors of CST efficacy.

**Discussion:**

Modified CST combined with medication significantly delays cognitive decline and improves QOL-AD in patients with mild-to-moderate AD. Educational attainment, lifestyle factors, and cognitive reserve are key determinants of CST efficacy. Relevant institutions should develop targeted enhancement protocols for patients with lower educational levels or insufficient cognitive reserves when implementing CST.

**Trial Registration:**

Chinese Clinical Trial Registry, identifier ChiCTR2600118654, https://www.chictr.org.cn.

## 1 Introduction

AD has become a major public health problem threatening the health of older adults worldwide (1). AD is the most common cause of dementia, accounting for approximately 60%–70% of all dementia cases according to the World Health Organization (2). With population aging, the burden of AD continues to increase rapidly. In China, a nationwide cross-sectional study estimated that the prevalence of AD among adults aged 60 years or older was 3.9%, corresponding to approximately 9.83 million affected individuals (3). In the United States, approximately 6.9 million people aged 65 years or older were living with AD in 2024, and this number is projected to increase substantially in the coming decades (4).

Current traditional drug therapies (such as cholinesterase inhibitors and memantine) exhibit limited efficacy, significant individual heterogeneity, and potential side effects (5). In addition to pharmacological treatment, non-pharmacological interventions are increasingly recognized as essential components of comprehensive AD management (6). These interventions include cognitive stimulation, cognitive training, reminiscence therapy, regular physical activity, music therapy, occupational therapy, caregiver education, and behavioral or environmental modification. Rather than directly targeting disease pathology, these approaches aim to preserve cognitive and functional abilities, promote social interaction, reduce behavioral and psychological symptoms, and improve quality of life. Among them, CST is one of the most widely studied psychosocial interventions for people with mild-to-moderate AD.

CST is an evidence-based psychosocial intervention that improves patients’ cognition and quality of life through structured group activities (7). CST is a structured, evidence-based psychosocial intervention developed by Spector and colleagues for people with mild-to-moderate dementia. The standard CST programme typically consists of 14 sessions delivered twice weekly over 7 weeks, with each session lasting approximately 45 minutes. It is commonly delivered in small groups through themed cognitive and social activities, such as reminiscence discussions, current affairs discussions, word association, object categorization, recognition tasks, number and word games, creative activities, and group competitions. These activities are designed to stimulate orientation, attention, memory, language, executive function, and social communication, while encouraging active engagement, verbal expression, and interpersonal interaction.

While evidence from Western populations confirms its efficacy, CST relies heavily on verbal communication and cultural contexts (8–10). Western protocols may not suit Chinese patients, and their specific benefits in this population require validation. Furthermore, studies have indicated individual variability in responses to CST (11). Currently, high-quality RCTs to identify factors that can independently predict treatment response and determine which patients may derive the greatest benefit from CST are lacking. This study aimed to evaluate the efficacy of modified CST as an adjunct to standard pharmacotherapy in patients with mild-to-moderate AD and to examine whether demographic and lifestyle characteristics could independently predict treatment response, thereby providing evidence-based support for personalized rehabilitation in AD.

## 2 Material and methods

### 2.1 Study design and ethics

**Figure 1:**
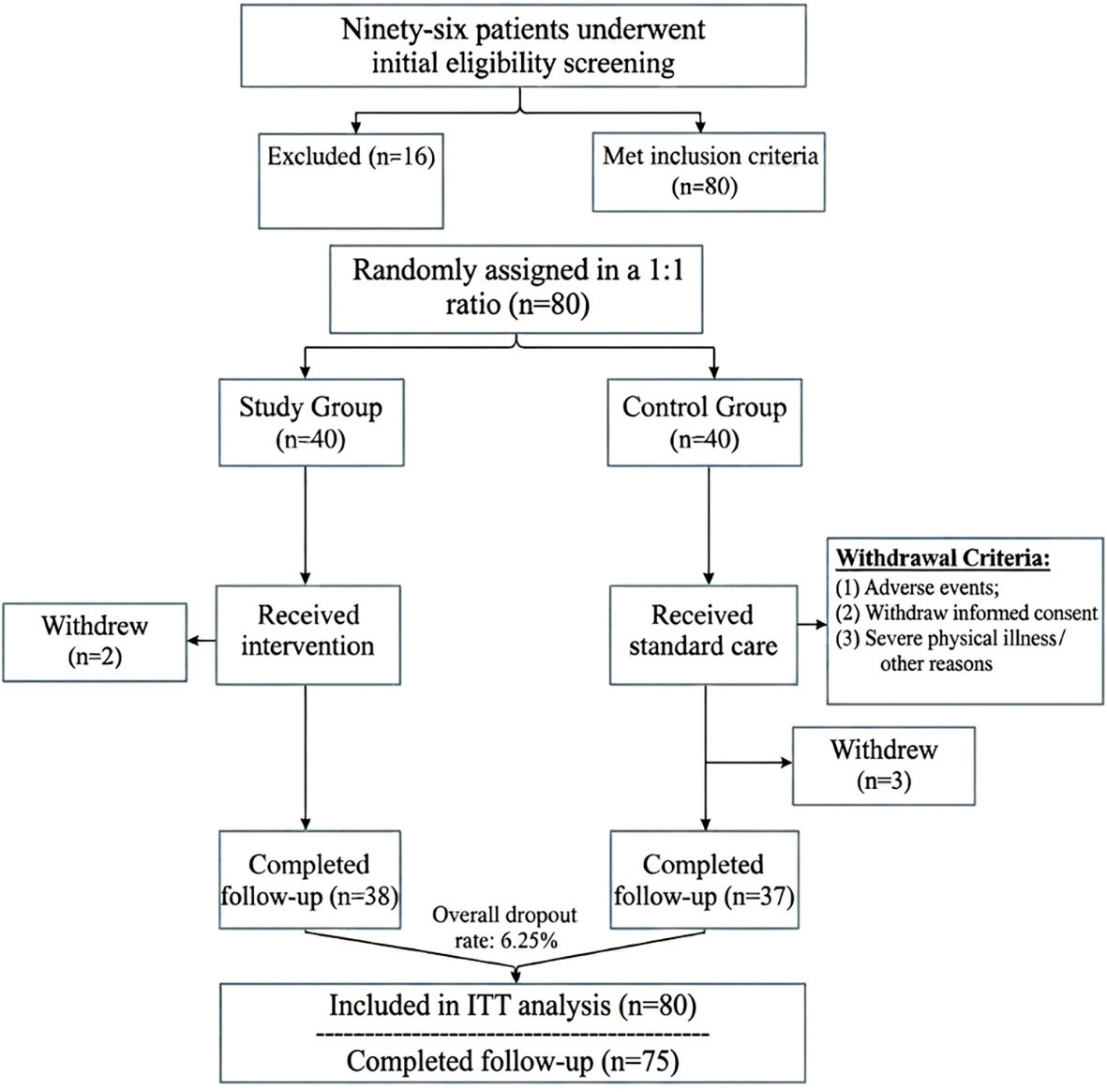
Consolidated standards of reporting trials (CONSORT) flow diagram.

This study was a single-center, evaluator-blinded, RCT conducted at the Geriatric Department of Chongqing Mental Health Center between January 2024 and October 2024. The overall participant recruitment, allocation, follow-up, and analysis process is shown in **Figure 1**.

As shown in **Figure 1**, 96 patients were screened for eligibility, of whom 80 were enrolled and randomized. Five participants discontinued the study, including two in the study group and three in the control group, resulting in an overall dropout rate of 6.25%. Seventy-five participants completed the study, and all 80 randomized participants were included in the ITT analysis.

This RCT was approved by the Ethics Committee of the Chongqing Mental Health Center (Approval No.: 2023 Lun Shen Yi Zi No. 019-1). The trial was retrospectively registered with the Chinese Clinical Trial Registry (Registration No.: ChiCTR2600118654). We confirm that this retrospective registration was due to an unintentional oversight, and that the study protocol had been prospectively approved by the Ethics Committee prior to the enrollment of the first participant. The study was conducted in strict accordance with the Declaration of Helsinki and the pre-approved protocol. All participants’ legal guardians provided informed consent. Raw data cannot be disclosed due to ethical restrictions, but aggregated datasets will be provided upon request (see the Data Availability Statement).

### 2.2 Study population

Elderly patients with AD admitted to the Geriatric Department of our center between January 2024 and October 2024 were selected.

#### 2.2.1 Inclusion criteria

The inclusion criteria were as follows: males and females aged 60–85 years (including 60 and 85 years); meeting the clinical diagnostic criteria for AD established by the National Institute on Aging-Alzheimer’s Association; Clinical Dementia Rating (CDR) score of 1.0 or 2.0, with MMSE score of 10–24; significant behavioral and psychological symptoms of dementia with Behavioral Pathology in Alzheimer’s Disease Rating Scale (BEHAVE-AD) score ≥ 8; no significant hearing or visual impairment; able to engage in simple communication and accept assessment after patient explanation; and no severe physical illness.

#### 2.2.2 Exclusion criteria

Patients were excluded if they had contraindications to cholinesterase inhibitors or antipsychotic medications or were otherwise deemed ineligible by the investigator.

### 2.3 Randomization and blinding

This study employed stratified block randomization and assigned participants in a 1:1 ratio to the study group, hereafter referred to as the study group (n = 40), or the control group, hereafter referred to as the control group (n = 40). Stratification was performed according to baseline cognitive status, defined as MMSE scores of 10–20 or 21–24, in order to balance dementia severity between the two randomized arms. Within each stratum, participants were randomly assigned using a block size of 4. Random sequences were generated using SAS 9.4 (IBM, Armonk, NY).

The random allocation sequence was managed by an independent statistician unrelated to the study and sealed in an opaque envelope. These were opened sequentially after the subjects had completed the baseline assessments and confirmed their eligibility. This stratification was applied at the allocation stage and did not necessarily determine the composition of individual intervention groups.

Due to the nature of the CST intervention, double blinding was not feasible for either the CST implementers or the subjects. This study employed a single-blind design. All outcome assessments (including the ADAS-Cog scale, ADL scale, and QOL-AD scale) were conducted by two third-party psychometricians who received standardized training and remained completely unaware of the participant groupings. These assessors did not participate in any intervention delivery or care during the study period and were explicitly instructed not to discuss treatment details with the participants to minimize measurement bias.

### 2.4 Intervention protocol

All participants received pharmacological treatment as prescribed by the attending clinicians in accordance with routine clinical practice. Participants with baseline MMSE scores of 21–24 received donepezil monotherapy, whereas those with MMSE scores of 10–20 received donepezil combined with memantine. Donepezil was initiated orally at 5 mg/day, increased to 10 mg/day at week 4, and maintained thereafter. Memantine was initiated at 5 mg/day and increased weekly by 5 mg to a maximum dose of 20 mg/day, as tolerated (12).

In addition to the above-mentioned medication regimen, participants in the study group received CST. The standard CST programme typically consists of 14 sessions delivered twice weekly over 7 weeks, with each session lasting approximately 45 minutes. It is commonly delivered in small groups through themed cognitive and social activities, such as reminiscence discussions, current affairs discussions, word association, object categorization, recognition tasks, number and word games, creative activities, and group competitions. These activities are designed to stimulate orientation, attention, memory, language, executive function, and social communication, while encouraging active engagement, verbal expression, and interpersonal interaction. However, high-frequency interventions demand greater physical stamina and long-term attendance commitment. Considering the physical tolerance and long-term adherence of elderly patients and referencing the favorable feasibility and cognitive benefits observed in Peak ‘s once-weekly, 7-session CST program for veterans (13), this study adjusted the standard CST frequency to once weekly for 45 min over 14 weeks.

### 2.5 CST content design

For practical delivery of the CST intervention, participants assigned to the study group were further organized into CST treatment groups of five to seven participants. These CST treatment groups were used only for the delivery of the group-based CST sessions and were not related to the randomization strata, medication regimen, or baseline MMSE category. Participants were allocated to CST treatment groups according to their availability, ward schedule, and feasibility of group interaction. The group size was limited to five to seven participants to facilitate communication, active participation, and individualized support during each session.

The entire program spanned 14 weeks, with each session lasting approximately 45 minutes. The course content was designed to account for cognitive variability among patients by incorporating sessions of varying difficulty levels to ensure that each participant progressed at an appropriate pace.

The program comprises three core processes: warm-up, themed, and closing activities. Each session began with a consistent warm-up segment, including ball-passing games and brief discussions on orientation questions (time, place, etc). This segment not only maintains session continuity but also helps patients enhance their orientation abilities. After approximately 10 minutes, the relaxed and enjoyable atmosphere sets a positive foundation for subsequent in-depth learning. Following the warm-up activities, each session featured a distinct theme from fourteen categories, including physical activities, sounds, childhood memories, food, current events, faces/landscapes, word association, creativity, object classification, orientation, money management, number of games, word games, and group competitions. This segment lasted for approximately 25 min. Finally, each session concluded with a summary and a single segment. The closing activities lasted for approximately 10 min.

The intervention was delivered by geriatric nurses from the research team who were designated as CST trainers. Prior to the start of the study, all CST trainers received formal and rigorous training in the CST protocol. This training included the theoretical principles of CST, the structure and procedures of each session, activity delivery, communication techniques, and procedures to ensure standardized implementation and intervention fidelity.

### 2.6 Assessment tools

This study employed internationally recognized standardized scales for evaluation, clearly defining the primary and secondary outcome measures. All assessments were performed at baseline (pre-intervention) and 14 weeks after the intervention.

#### 2.6.1 Primary outcome measure

The primary endpoint was the change in the ADAS-Cog score from baseline to week 14. The ADAS-cog is one of the gold standards for assessing cognitive impairment and treatment responses in patients with AD, comprising 11 tasks covering memory, language, and operation/praxis. The total score ranges from 0 to 70 and is calculated as the sum of errors across all items, with higher scores indicating more severe cognitive impairment (14).

#### 2.6.2 Secondary outcome measures

Secondary endpoints included changes in ADL and QOL-AD scores from baseline to week 14.

The QOL-AD scale is used to evaluate the quality of life of patients with AD. It consists of 13 items scored from 1 to 4 points, yielding a total score of 13 to 52 points. Higher scores indicate a better quality of life, while lower scores indicate a poorer quality of life (15).

The ADL scale is comprised of two components: the Physical ADL Scale and the Instrumental ADL Scale, consisting of 14 items with a total score ranging from 0 to 64. This is a reverse-scoring scale (lower scores indicate better functioning) (16).

### 2.7 Screening and stratification tools

The MMSE assesses cognitive impairment in patients and is a crucial screening tool for evaluating overall cognitive function and impairment severity in AD. Higher scores indicate better cognitive function (17).

The CDR Scale was used to evaluate the severity of overall cognitive and daily living function impairment in subjects, where 1, 2, and 3 points indicate mild, moderate, and severe dementia, respectively (18).

### 2.8 Statistical analysis

Based on the effect size (Cohen’s d = 0.81) of the ADAS-Cog from the pilot study, sample size estimation for intergroup comparisons was performed using GPower 3.1 software. Under conditions of α=0.05 (two-tailed) and β=0.20 (80% test power), a minimum of 38 subjects per group was calculated. The actual enrollment of 80 patients (40 per group) met the pre-specified statistical power requirement (19).

Data were processed using SPSS 26.0 (IBM). Quantitative data are expressed as the mean ± standard deviation. Intergroup comparisons were performed using independent-sample t-tests or Mann–Whitney U tests. Categorical data were analyzed using χ^2^ tests. Efficacy analysis followed the ITT principle, with missing cases imputed using the worst-case scenario. Specifically, for participants in the CST group with missing post-intervention data, the least favorable observed change among participants with available data was imputed. For participants in the control group with missing post-intervention data, the most favorable observed change among participants with available data was imputed.

The primary outcome was the post-intervention ADAS-Cog score. To account for baseline differences and improve statistical precision, between-group differences in post-intervention ADAS-Cog scores were analyzed using analysis of covariance, with the post-intervention ADAS-Cog score as the dependent variable, treatment group as the fixed factor, and baseline ADAS-Cog score as the covariate. The adjusted mean difference between groups and its 95% confidence interval were reported. A lower ADAS-Cog score indicates better cognitive performance. Secondary outcomes, including ADL and QOL-AD scores, were analyzed using the same baseline-adjusted ANCOVA approach, with the corresponding post-intervention score as the dependent variable, treatment group as the fixed factor, and the corresponding baseline score as the covariate.

To identify predictors of CST efficacy (ADAS-Cog improvement scores) while ensuring statistical power with a limited sample size, a two-stage screening strategy was employed. Potential predictors associated with response to CST were explored using a two-stage analytic strategy consisting of univariate screening followed by stepwise multivariable regression. Statistical significance was set at P < 0.05.

## 3 Results

### 3.1 Baseline characteristics

A total of 80 patients with mild-to-moderate AD were randomized, with 40 assigned to the study group and 40 assigned to the control group. During the 14-week intervention period, five participants discontinued the study, including two in the study group and three in the control group. Therefore, 75 participants completed the study. All 80 randomized participants were included in the ITT. The baseline demographic and clinical characteristics of the participants are shown in **Table 1**. There were no statistically significant differences between the study and control groups in age, sex distribution, disease duration, years of education, baseline MMSE score, dementia severity, medication regimen, regular physical activity, comorbidities, or active hobbies (all P > 0.05), indicating that the two groups were comparable at baseline. Participants with baseline MMSE scores of 21–24 received donepezil monotherapy, whereas those with baseline MMSE scores of 10–20 received donepezil plus memantine.

**Table 1:** Comparison of age, sex, disease duration, years of education, baseline MMSE score, comorbidities, hobby engagement, regular physical activity, dementia severity, and medication regimen between groups.

Variable		Study group (*n* = 40)	Control group (*n* = 40)	*T*/*χ*^2^ *value*	*P value*
Age (years)	65-69	3	4	2.858	0.582
	70-74	4	9		
	75-79	22	18		
	80-84	9	8		
	≧85	2	1		
Sex	Male	16	19	0.457	0.499
	Female	24	21		
Disease duration (months)		47.6 ± 16.2	43.9± 11.1	1.208	0.231
Years of education (years)	0-6	4	6	0.600	0.741
	7-12	11	9		
	>12	5	5		
Baseline MMSE score		16.1 ± 2.6	16.9 ± 2.6	-1.39	0.169
Comorbidities	Yes	24	22	0.205	0.651
	no	16	18		
Hobby engagement	active	22	20	0.202	0.654
	Inactive	18	20		
Regular physical activity	30 minutes or more	12	10	0.300	0.861
	Less than 30 minutes	16	18		
	Rare physical activity	12	12		
Dementia severity	Mild	6	8	0.34	0.556
	Moderate	34	32		
Medication regimen	Donepezil monotherapy	6	8	0.34	0.556
	Donepezil plus memantine	34	32		

Data are presented as the mean ± standard deviation for continuous variables or n for categorical variables. Between-group comparisons were performed using independent-samples t tests, chi-square tests, or Fisher’s exact tests, as appropriate.

### 3.2 Cognitive function improvement

After 14 weeks of intervention, ADAS-Cog scores decreased from baseline in both groups, indicating an improvement in cognitive performance. The decrease was statistically significant in the study group, with scores decreasing from 36.2 ± 3.1 to 30.6 ± 2.8, corresponding to a mean change of -5.6 ± 1.2 points (t = 6.324, P < 0.001). In the control group, ADAS-Cog scores decreased from 36.0 ± 3.0 to 33.7 ± 2.9, with a mean change of -2.3 ± 0.9 points; however, this within-group change was not statistically significant (t = 1.715, P = 0.094). Intergroup comparison showed that the improvement in ADAS-Cog scores was significantly greater in the study group than in the control group, with a mean difference of -3.3 points (95% CI: -4.1, -2.5; t = -2.52; P = 0.008). Effect size analysis showed a Cohen’s d of 0.81, indicating a moderate-to-large treatment effect (**Table 2**).

**Table 2:** Baseline-adjusted comparison of changes in ADAS-Cog scores between the study and control groups.

Group	n	Pre-intervention mean ± SD	Post-intervention mean ± SD	Mean change mean ± SD	Adjusted mean difference	95% CI	F	P value
Study Group	40	36.2 ± 3.1	30.6 ± 2.8	-5.6 ± 1.2	-3.28	(-3.72, -2.83)	214.3	<0.001
Control Group	40	36.0 ± 3.0	33.7 ± 2.9	-2.3 ± 0.9	Reference	—	—	—

Data are presented as mean ± standard deviation. The mean change was calculated as the post-intervention score minus the pre-intervention score. Between-group comparisons were performed using analysis of covariance, with the post-intervention ADAS-Cog score as the dependent variable, treatment group as the fixed factor, and baseline ADAS-Cog score as the covariate. The control group was used as the reference category in the ANCOVA model.

### 3.3 ADL and QOL-AD

After 14 weeks of intervention, the study group showed significantly greater improvement than the control group in both QOL-AD and ADL scores. For QOL-AD, the study group showed a mean increase of 4.77 ± 1.62 points, whereas the control group showed a mean increase of 2.08 ± 0.97 points. Baseline-adjusted ANCOVA showed a significant between-group difference, with an adjusted mean difference of 2.69 points (95% CI: 1.01 to 4.37; F = 10.19; P = 0.002). For ADL, the study group showed a mean decrease of 8.35 ± 3.11 points, whereas the control group showed a mean decrease of 3.42 ± 2.54 points. The adjusted mean difference was -4.93 points (95% CI: -8.39 to -1.47; F = 8.03; P = 0.006), indicating significantly greater improvement in the study group (**Table 3**).

**Table 3:** Baseline-adjusted comparison of changes in QOL-AD and ADL scores between the study and control groups.

Outcome	Group	Baseline mean ± SD	Post-intervention mean ± SD	Change mean ± SD	Adjusted mean difference	95% CI	F	P value
QOL-AD	Study Group	28.2 ± 3.0	33.0 ± 2.8	+4.77 ± 1.62	+2.69	(1.01, 4.37)	10.19	0.002
	Control Group	29.4 ± 2.9	31.5 ± 2.7	+2.08 ± 0.97	Reference			
ADL	Study Group	33.6 ± 3.4	25.3 ± 3.1	−8.35 ± 3.11	−4.93	(−8.39 −1.47)	8.03	0.006
	Control Group	31.0 ± 3.2	27.6 ± 3.0	−3.42 ± 2.54	Reference			

Data are presented as mean ± SD. Between-group comparisons were performed using ANCOVA, with the post-intervention score as the dependent variable, treatment group as the fixed factor, and the corresponding baseline score as the covariate. The adjusted mean difference represents the baseline-adjusted difference between the study group and the control group. CI, confidence interval.

### 3.4 Analysis of predictors of treatment effectiveness

To explore key factors influencing CST efficacy, we conducted univariate analyses of baseline characteristics—including age, sex, years of education, baseline MMSE, disease duration, comorbidities, regular physical activity, and hobby engagement. Using ADAS-Cog improvement values (Δ values) in the study group as the dependent variable. Preliminary screening revealed no significant association between efficacy and sex, age, or comorbidities (all P > 0.2); thus, these variables were excluded from subsequent models. After incorporating years of education, regular physical activity, baseline MMSE scores, and hobby engagement into a multiple linear regression model and eliminating confounding factors through stepwise regression analysis, in the final multivariable linear regression model, education, hobby engagement, regular physical activity, and baseline MMSE score remained significantly associated with changes in ADAS-Cog scores. Higher education level was associated with greater reductions in ADAS-Cog scores (β = -0.536, t = -5.936, P < 0.001). Active hobby engagement (β = -0.198, t = -3.304, P = 0.002), regular physical activity (β = -0.278, t = -3.373, P = 0.002), and a higher baseline MMSE score (β = -0.215, t = -3.707, P = 0.001) were also associated with greater reductions in ADAS-Cog scores. The final model was statistically significant, explaining 67.1% of the variance in ADAS-Cog score changes, with R^2^ = 0.671, adjusted R^2^ = 0.633, F (4, 35) = 17.82, P < 0.001 (**Table 4**).

**Table 4:** Exploratory multivariate analysis of predictors of ADAS-Cog score changes in the study group.

Variables	SE	*β*	t-value	P value
Years of education	0.063	-0.536	-5.936	<0.001
Hobby engagement	0.104	-0.198	-3.304	0.002
Regular physical activity	0.081	-0.278	-3.373	0.002
Baseline MMSE	0.058	-0.215	-3.707	0.001

Model summary: R^2^ = 0.671, adjusted R^2^ = 0.633, F (4, 35) = 17.82, P < 0.001.

The predictor analysis was exploratory and was conducted only within the intervention group. The dependent variable was the change in the ADAS-Cog score after the 14-week intervention. Negative β values indicate greater reductions in ADAS-Cog scores and therefore greater cognitive improvement. The final multivariable linear regression model included education, hobby engagement, regular physical activity, and baseline MMSE score. The model was statistically significant, with R^2^ = 0.671, adjusted R^2^ = 0.633, F (4, 35) = 17.82, P < 0.001. Education and baseline MMSE score were treated as continuous variables. Hobby engagement was coded as inactive = 0 and active = 1. Regular physical activity was coded as rarely regular physical activitys = 0, less than 30 minutes per day = 1, and 30 minutes or more per day = 2. Presence of comorbidities was included as a candidate variable and was coded as no = 0 and yes = 1.

Because this predictor analysis was restricted to the intervention group, used a relatively small sample size, and applied univariate screening followed by stepwise regression, the findings should be considered exploratory and interpreted with caution due to the potential risk of overfitting.

## 4 Discussion

This RCT examined the efficacy of modified CST combined with medication in patients with mild-to-moderate AD and further explored factors associated with treatment response. Compared with medication alone, the combined intervention produced greater improvement in cognitive function, as measured by the ADAS-Cog, and was also associated with greater improvement in activities of daily living and quality of life. The observed effect size for cognitive improvement suggests that the benefit was not limited to statistical significance but may also have clinical relevance. In the exploratory regression analysis, years of education, leisure activity participation, regular physical activity, and baseline cognitive status were associated with the magnitude of improvement after CST. These results indicate that modified CST may be a useful adjunct to pharmacological treatment, while also suggesting that patient characteristics should be considered when interpreting and applying this intervention.

The cognitive findings are in line with previous studies reporting beneficial effects of CST and other structured cognitive interventions in people with dementia (20). CST differs from simple cognitive physical activities in that it combines orientation, memory, language, attention, problem-solving, reminiscence, and interpersonal communication within a structured group format (21). This multidomain design may encourage patients to repeatedly use preserved cognitive abilities and compensatory strategies in a socially meaningful context (22). In the present trial, the better ADAS-Cog outcome in the study group suggests that such structured stimulation may add benefit to standard medication. However, because the intervention contained several interacting components, the current data cannot determine whether the improvement was mainly driven by cognitive training, group interaction, therapist guidance, or their combined effects. These findings should therefore be understood as evidence for the overall effectiveness of the modified CST package rather than for any single active component.

A clinically plausible explanation for the superior outcome in the combined-treatment group is that medication and CST may have complementary roles (23). Cholinesterase inhibitors, such as donepezil, increase synaptic acetylcholine availability by inhibiting acetylcholinesterase activity and are widely used to support cholinergic neurotransmission in AD (24). CST, by contrast, provides repeated opportunities for cognitive engagement, communication, and task-oriented participation. Patients receiving stable pharmacological treatment may therefore be better positioned to benefit from structured cognitive stimulation. This interpretation is consistent with the broader dementia literature, suggesting that combined pharmacological and non-pharmacological approaches may produce more favorable outcomes than either strategy alone (25).

An important finding of this trial is that the observed benefit was not confined to cognitive test performance. Patients in the CST group also showed greater improvement in ADL and QOL-AD, which may reflect the broader therapeutic context of group-based CST. Medication primarily targets cognitive symptoms, whereas CST provides social contact, emotional support, communication practice, and opportunities for meaningful participation (26). These elements may influence mood, self-confidence, motivation, and willingness to engage in daily activities. Previous research has linked social engagement with better cognitive and emotional outcomes in older adults, while loneliness and social isolation have been associated with poorer prognosis in dementia (27). In this sense, the improvement in QOL-AD scores may partly reflect non-specific but clinically important effects of social interaction and group participation. This is not necessarily a weakness of CST; rather, it highlights that psychosocial engagement may be one of the pathways through which CST produces clinically meaningful benefit.

The regression analysis suggested that educational attainment was one of the strongest predictors of response. This finding is compatible with the cognitive reserve hypothesis, which proposes that individuals with higher education may be better able to cope with AD-related neuropathology through more efficient or flexible use of cognitive networks (28). In a CST setting, higher educational attainment may also make it easier for patients to understand task instructions, participate in discussions, and apply cognitive strategies. Nevertheless, education is an imperfect proxy for cognitive reserve. It may also be associated with socioeconomic status, lifelong cognitive activity, health literacy, and access to care (29). Therefore, although the association between education and CST response is clinically meaningful, it should not be interpreted as direct evidence that cognitive reserve was the causal mechanism.

Leisure activity participation was another factor associated with greater cognitive improvement. This result is relevant because leisure activities may represent a current and modifiable source of cognitive and social engagement (30). Unlike years of education, which mainly reflect early-life and mid-life enrichment, hobbies and leisure participation indicate ongoing stimulation in later life. Patients who continue to engage in reading, games, music, social activities, gardening, or other hobbies may have better preserved initiative and functional capacity, making them more responsive to structured intervention (31). Prior studies have reported associations between cognitively and socially stimulating activities and better cognitive outcomes in older adults (32). However, leisure activity may also be a marker of less severe disease, stronger family support, better mood, or higher motivation. More detailed assessment of leisure activity type, frequency, duration, and cognitive demand would be needed to clarify its role in CST responsiveness.

Regular physical activity was also associated with treatment response. Regular physical activity has been linked to cognitive and functional outcomes through several possible pathways, including vascular health, sleep quality, mood regulation, inflammation, and neurotrophic signaling (33). Some studies have reported associations between regular physical activity, brain-derived neurotrophic factor, and hippocampal function, which are relevant to learning and memory (34). In the present study, however, regular physical activity was assessed as a behavioral habit rather than through objective measures of physical fitness or biological markers. A cautious interpretation is therefore warranted. Regular physical activities may have contributed to a physiological state more favorable for learning, but they may also simply identify patients with better general health, stronger adherence, or greater behavioral activation. Future trials that combine CST with structured regular physical activity programs would be useful for determining whether these two interventions have additive or synergistic effects.

Baseline cognitive status also appeared to influence the magnitude of benefit. Patients with higher baseline MMSE scores may have retained better attention, comprehension, communication ability, and task-learning capacity, all of which could facilitate participation in CST. This finding supports the clinical view that non-pharmacological interventions may be particularly valuable when introduced during the earlier stages of cognitive decline (35). However, it should not be taken to mean that patients with lower baseline cognition are unsuitable for CST. Instead, these patients may need adapted formats, such as simpler materials, greater repetition, shorter tasks, smaller groups, or greater caregiver involvement. Matching task difficulty to remaining cognitive capacity may be essential for maintaining engagement and avoiding frustration.

Clinically, the present findings support the use of modified CST as an adjunct to standard medication for patients with mild-to-moderate AD. The value of CST may lie in its combined cognitive, social, emotional, and functional components. The predictor findings also suggest that a uniform CST protocol may not be optimal for all patients. Individuals with lower educational attainment, limited leisure engagement, sedentary lifestyles, or more impaired baseline cognition may require additional support or adjustments in intervention format or intensity. These considerations may help clinicians tailor CST more appropriately. However, because the predictor analysis was exploratory, such stratified approaches should be tested prospectively before being adopted as formal clinical recommendations.

Several limitations should be noted. First, the sample size was relatively small, and the regression analysis was conducted only in the intervention group. Given the number of candidate predictors, the model may be unstable and vulnerable to overfitting. The predictor results should therefore be considered hypothesis-generating. Second, the control group received medication alone and did not receive an active social-contact control condition. As a result, the study cannot fully separate the specific effects of cognitive stimulation from the effects of therapist attention, expectancy, social interaction, and group participation. Third, the follow-up period was limited, so the durability of the observed cognitive and functional benefits remains uncertain. Larger trials with longer follow-up and active control conditions are needed to confirm the robustness and specificity of these findings.

Future research should further examine the mechanisms through which CST exerts its effects. Functional magnetic resonance imaging (fMRI) could help determine whether CST is associated with changes in functional connectivity or task-related activation patterns (36). Biomarkers such as brain-derived neurotrophic factor or inflammatory markers may clarify whether clinical improvement is accompanied by measurable biological change (37). Incorporating these measures, together with more detailed behavioral assessments, would allow future studies to distinguish cognitive, psychosocial, and biological pathways of response. Such evidence would be valuable for developing more personalized and methodologically grounded CST protocols for patients with AD.

## 5 Conclusion

This RCT showed that modified CST combined with medication produced greater improvements in cognitive function, ADL, and QOL-AD than medication alone in patients with mild-to-moderate AD. The exploratory analyses suggested that educational attainment, leisure activity participation, regular physical activity, and baseline cognitive status may influence treatment response. These findings support modified CST as a useful adjunctive intervention in dementia care, but the predictor results should be interpreted cautiously because of the limited sample size. Further studies with larger samples, active control conditions, longer follow-up, and mechanistic assessments are needed to confirm these findings and guide more individualized CST protocols.

## Data Availability

The raw data supporting the conclusions of this article will be made available by the authors, without undue reservation.
